# Routine Laboratory Results and Thirty Day and One-Year Mortality Risk Following Hospitalization with Acute Decompensated Heart Failure

**DOI:** 10.1371/journal.pone.0012184

**Published:** 2010-08-17

**Authors:** Victor Novack, Michael Pencina, Doron Zahger, Lior Fuchs, Roman Nevzorov, Allan Jotkowitz, Avi Porath

**Affiliations:** 1 Clinical Research Center, Soroka University Medical Center, Beer-Sheva, Israel; 2 Harvard Clinical Research Institute, Boston, Massachusetts, United States of America; 3 Cardiology, Soroka University Medical Center, Beer-Sheva, Israel; Lerner Research Institute, United States of America

## Abstract

**Introduction:**

Several blood tests are performed uniformly in patients hospitalized with acute decompensated heart failure and are predictive of the outcomes: complete blood count, electrolytes, renal function, glucose, albumin and uric acid. We sought to evaluate the relationship between routine admission laboratory tests results, patient characteristics and 30-day and one-year mortality of patients admitted for decompensated heart failure and to construct a simple mortality prediction tool.

**Methods:**

A retrospective population based study. Data from seven tertiary hospitals on all admissions with a principal diagnosis of heart failure during the years 2002–2005 throughout Israel were captured.

**Results:**

8,246 patients were included in the study cohort. Thirty day mortality rate was 8.5% (701 patients) and one-year mortality rate was 28.7% (2,365 patients). Addition of five routine laboratory tests results (albumin, sodium, blood urea, uric acid and WBC) to a set of clinical and demographic characteristics improved c-statistics from 0.76 to 0.81 for 30-days and from 0.72 to 0.76 for one-year mortality prediction (both p-values <0.0001). Three dichotomized abnormal laboratory results with highest odds ratio for one-year mortality (hypoalbuminaemia, hyponatremia and elevated blood urea) were used to construct a simple prediction score, capable of discriminating from 1.1% to 21.4% in 30-day and from 11.6% to 55.6% in one-year mortality rates between patients with a score of 0 (1,477 patients) vs. score of 3 (544 patients).

**Discussion:**

A small set of abnormal routine laboratory results upon admission can risk-stratify and independently predict 30-day and one-year mortality in patients hospitalized with acute decompensated heart failure.

## Introduction

Heart failure (HF) is a major worldwide public health concern. Currently, more than 5 million people in the United States are diagnosed with HF, and approximately 550,000 new cases are reported annually with an estimated annual cost of $33.2 billion.[1]

Several blood tests are performed almost uniformly in patients hospitalized with acute, decompensated heart failure: a complete blood count, electrolytes levels, renal function, glucose, albumin and uric acid levels. Anemia is a powerful independent predictor of death and recurrent hospitalization in different subclasses of HF.[2] Single measurement of the total white blood cells (WBC) count is associated with all-cause and cardiovascular mortality in clinically stable patients with left ventricular (LV) systolic dysfunction.[3], [4] Renal impairment is a frequent prognosticator for mortality in patients hospitalized with HF.[5], [6] Hyponatremia and hyperkalemia are common in patients admitted with heart failure and have been shown to predict mortality.[7], [8], [9], [10] Uric acid (UA) levels can be a marker of impaired prognosis in hospitalized patients with acute HF and LV systolic dysfunction.[11], [12] Low albumin concentration can be an indicator of inflammation superimposed on cardiac cachexia – both factors associated with increased mortality in populations with HF.[13], [14] Finally, high blood glucose levels are associated with worsening of HF in diabetic and non-diabetic subjects.[15], [16]

Lately numerous new biomarkers were evaluated as prognosticators in HF population.[17], [18], [19] However, the limited availability of these tests and lack of the standardization makes their use problematic in day-to-day clinical practice. We hypothesize that routine laboratory tests can be used for the same purpose, i.e. prediction of outcome in patients hospitalized with acute HF.

The purpose of this study was to construct and validate a predictive tool for 30-day and one-year all cause mortality based on a minimal set of admission routine laboratory tests together with basic patient data in a nationally representative group of patients hospitalized for acute heart failure.

## Methods

### Ethics Statement

The study was a part of a quality control project. For the purposes of the analysis deidentified database was created from the hospitals administrative databases. Therefore no Ethics Committee approval or subject informed consent was needed.

All heart failure admissions to the seven major general hospitals of the Clalit Health Services throughout Israel (30% of general hospitals beds in the country) from November 2001 to June 2005 were screened. Each of the seven general hospitals transfers patient level data to a central data warehouse, which is connected to the computerized database of the Interior Ministry. The latter provides real life information on the vital status of citizens. Patients are identified in all databases by the unique national ID number. The medical records of patients with primary discharge diagnosis consistent with HF (*International Classification of Diseases, Ninth Revision* code 428) and no concurrent myocardial infarction were assessed electronically. For a sub-cohort of Clalit insured patients (approximately 80% of the cohort), data on pre-hospitalization medications were obtained.

We assessed the first available results of the laboratory test performed within first 24 hours of admission. Based on *predefined definitions* based on limits of normal range and the assessment of the relationships between laboratory values and one-year mortality we dichotomized the following variables: sodium (hyponatremia <136 meq/L), urea (elevated ≥43 mg/dL), anemia (hematocrit <40% in men and <36% in women), albumin (low <3.5 g/dL), uric acid (elevated >6.5 mg/dL) and glucose (elevated≥200 mg/dL) and trichotomized white blood cells count (WBC leukocytosis >10.8 10^3^/mm^3^, leucopenia<4.3 10^3^/mm^3^) and potassium (hyperkalemia >5.0 meq/L, hypokalemia<3.5 meq/L). Glomerular filtration rate (GFR) was estimated based on the Modification of Diet in Renal Disease (MDRD) Study equation and dichotomized into two groups: ≥60 ml/min and <60 ml/min. We used the Charlson index to compute the burden of comorbid conditions.[20]

### Statistical Analysis

The primary outcomes were 30-day and 1 year all cause deaths. For univariate analysis, we used t-test for comparison of continuous variables, and Pearson's chi-square test for categorical variables. Mann-Whitney test was used for comparison of variables with non-normal distribution.

Risk prediction models were developed for 30-day and one-year mortality. Since full follow-up was available on all participants and primary interest was on event occurrence rather than its timing, logistic regression was employed. Variable selection in multivariable modeling was based on clinical and statistical significance and performed in a hierarchical fashion, following the approach outlined in.[21] In case of pair-wise correlations between laboratory tests exceeding 0.50, we chose only one of those tests: the one with stronger individual effect size (this led to selecting urea over GFR and RBC over hematocrit).

First, all laboratory tests results significantly associated with one-year mortality in univariate analysis were included into the regression models for 30-day and one-year death as continuous variables. Since WBC and potassium levels had non-linear relationship with one-year mortality, those variables were included in a categorical form (three groups): below low limit of normal, normal (reference group) and above upper limit of normal. Multiple partial tests were performed to confirm their significance as a group. At the next step baseline clinical and demographic characteristics: age, sex, history of myocardial infarction, dyslipidemia, arterial hypertension, diabetes, history of bypass surgery, atrial fibrillation, chronic obstructive pulmonary disease, dementia and Charlson index were added. Finally pre-hospitalization medications were introduced: angiotensin-converting enzyme inhibitors, angiotensin receptors blockers, β-blockers, statins, spironolactone, digoxin and diuretics and tests of significance for laboratory variables were repeated.

To investigate the most appropriate functional form (linear, quadratic etc.) of the relationship between laboratory tests and one-year mortality we fit generalized additive models which allow for modeling the predictors as spline functions thus relaxing the assumption of linearity (SAS proc GAM).[22] The results confirmed those obtained by crudely plotting event rates across sub-categories of laboratory tests.

Discriminatory abilities of the models were assessed by area under the receiver operating characteristics (ROC) curves (AUC) for death probability calculated for each subject. Discriminatory abilities of the models were compared using a test for difference of two AUCs proposed by DeLong et al. and available through the roccontrast option in SAS proc logistic. [23] Calibration of the models was evaluated by Hosmer and Lemeshow chi-square test of goodness of fit. For model derivation we randomly selected 3 out of 7 hospitals. Once the models were derived and shown to be valid in the other 4 hospitals, the two cohorts (derivation and validation) were combined for further analysis.

To further evaluate the models' performance the echocardiography data obtained within ±3 month from the index hospitalization for 854 subjects admitted to one of the hospital were assessed. Ejection fraction and presence of diastolic dysfunction were included into the models predicting 30-day and 1 year mortality and c-statistics were calculated.

To simplify clinical application the final model was repeated with significant laboratory test results dichotomized or trichotomized as described earlier and three of those variables with the highest odds ratios for one-year mortality were selected for inclusion into the clinical score. We assigned 1 point for each pathological result. Combined score was calculated for each patient. Kaplan-Meier analysis with log-rank test was used for assessing the relationship between the combined score and one-year mortality. All reported P values are two-sided and p<0.05 was considered significant.

## Results

### Study population

During 44 months of the study 15,661 admissions to seven hospitals with a principal diagnosis of heart failure decompensation were recorded 45.2% of which were repeat admissions. Overall 8,246 patients with a first HF admission during the study period comprised the study cohort. Median hospitalization duration was 4 days (interquartile range [IQR] of 2 to 7 days). During the hospital stay 467 patients (5.7%) died. Thirty day mortality rate was 8.5% (701 patients) and one-year mortality rate was 28.7% (2,365 patients).

Table 1 depicts baseline characteristics of the patient population stratified by one-year mortality. The deceased patients compared with the one year survivors were older (79.4%±10.9% vs. 74.0%±11.8%; p<0.001), and had more comorbidities as indicated by higher Charlson Comorbidity index (5.3%±1.8% vs. 4.3%±1.7%;p<0.001). Although the deceased patients had lower prevalence of diabetes mellitus (35.3% vs. 39.5%; p<0.001), hypertension (53.6% vs. 61.2%; p<0.001), dyslipidemia (18.6% vs. 29.1%;p<0.001) and previous CABG surgery (13.8% vs. 17.6%), they had higher prevalence of atrial fibrillation (40.3% vs. 34.2%), valvular diseases (15.0% vs. 11.1%), acute and chronic renal failure (8.2% vs. 3.3% and 21.4% vs. 11.8% respectively), anemia (27.6% vs. 22.0%), pleural effusion or ascites (10.3% vs. 7.0%), chronic obstructive pulmonary disease (16.8% vs.13.2%) and much higher prevalence of dementia (9.4% vs. 2.9%, all with p-value <0.001). Of note, survivors had higher rates of life saving cardiac medications use, such as ACE inhibitors (10.1% vs. 7.1%), β-blockers (59.1% vs.50.0%), statins (42.7% vs. 29.8%) and aspirin(54.1% vs.48.7%) but lower rates of spironolacton (18.4% vs. 23.4%) and diuretics use(73.9% vs. 78.8%, all with p-value <0.001). All laboratory values at admission (except glucose levels) were significantly different between survivors and deceased.

**Table 1:** Baseline characteristics of the patient population.

	All patients	One-year survivors	Deceased	p-value
	N = 8,246	N = 5,881	N = 2,365	
**Age, year**	75.6±11.8	74.0±11.8	79.4±10.9	<0.001
**Gender, female (%)**	47.8	47.1	49.6	0.04
**Charlson score, (points)**	4.6±1.8	4.3±1.7	5.3±1.8	<0.001
**Co-morbidities**				
Ischemic heart disease, (%)	60.8	61.4	59.4	0.10
Previous myocardial infarction, (%)	24.2	24.2	24.4	0.84
Diabetes, (%)	38.3	39.5	35.3	<0.001
Arterial hypertension, (%)	59.0	61.2	53.6	<0.001
Dyslipidemia, (%)	26.1	29.1	18.6	<0.001
Atrial fibrillation, (%)	35.9	34.2	40.3	<0.001
Non ischemic cardiomyopathy, (%)	4.1	4.2	3.7	0.25
Previous CABG, (%)	16.5	17.6	13.8	<0.001
Previous valvular surgery, (%)	3.3	3.4	3.1	0.49
Any valvular disease, (%)	27.7	27.5	28.2	0.47
Aortic valve disease, (%)	12.2	11.1	15.0	<0.001
Mitral valve disease, (%)	18.5	19.1	17.1	0.03
Chronic renal failure, (%)	14.5	11.8	21.4	<0.001
Acute renal failure, (%)	4.8	3.3	8.2	<0.001
Anemia, (%)	23.6	22.0	27.6	<0.001
Pleural effusion or ascites, (%)	7.9	7.0	10.3	<0.001
Chronic obstructive pulmonary disease, (%)	14.2	13.2	16.8	<0.001
Dementia, (%)	4.7	2.9	9.4	<0.001
**Medications at 3 months prior to hospitalization (n = 7,105)**				
Angiotensin-converting enzyme inhibitors, (%)	59.9	60.6	58.0	0.04
Angiotensin receptor blockers, (%)	9.3	10.1	7.1	<0.001
Aspirin, (%)	52.5	54.1	48.7	<0.001
β-blockers, (%)	56.5	59.1	50.0	<0.001
Statins, (%)	39.0	42.7	29.8	<0.001
Spironolactone, (%)	19.8	18.4	23.4	<0.001
Digoxin, (%)	15.1	14.6	16.4	0.05
Diuretics, (%)	75.3	73.9	78.8	<0.001

### Laboratory analysis (Table 2)

**Table 2:** Baseline laboratory characteristics of the patient population.

	All patients	One-year survivors	Deceased	p-value
	N = 8,246	N = 5,881	N = 2,365	
**Laboratory values at admission**				
Hematocrit, % (n = 8,047)	37.1±5.7	37.5±5.7	36.2±5.8	<0.001
RBC, 10^3^/mm^3^ (n = 8,065)	4.29±0.71	4.35±0.69	4.15±0.73	<0.001
WBC, 10^3^/mm^3^ (n = 8,064)	9.8±4.6	9.5±4.1	10.3±5.5	<0.001
Blood urea, (n = 8,034)	62.7±37.4	56.5±31.6	78.0±45.5	<0.001
Glomerular filtration rate, ml/min (n = 7,685)	42.3±19.0	44.6±18.6	36.8±18.6	<0.001
Sodium, mEq/L (n = 7,963)	137.3±4.8	137.6±4.5	136.4±5.3	<0.001
Potassium, mEq/L (n = 7,601)	4.5±0.7	4.5±0.6	4.6±0.8	<0.001
Albumin, g/dL (n = 7,775)	3.6±0.5	3.7±0.5	3.4±0.5	<0.001
Glucose, mg/dL (n = 8,028)	162.7±80.6	162.2±79.4	164.0±83.3	0.38
Uric acid, mg/dL (n = 6,755)	8.0±2.6	7.7±2.3	8.8±3.0	<0.001

**Figure 1:**
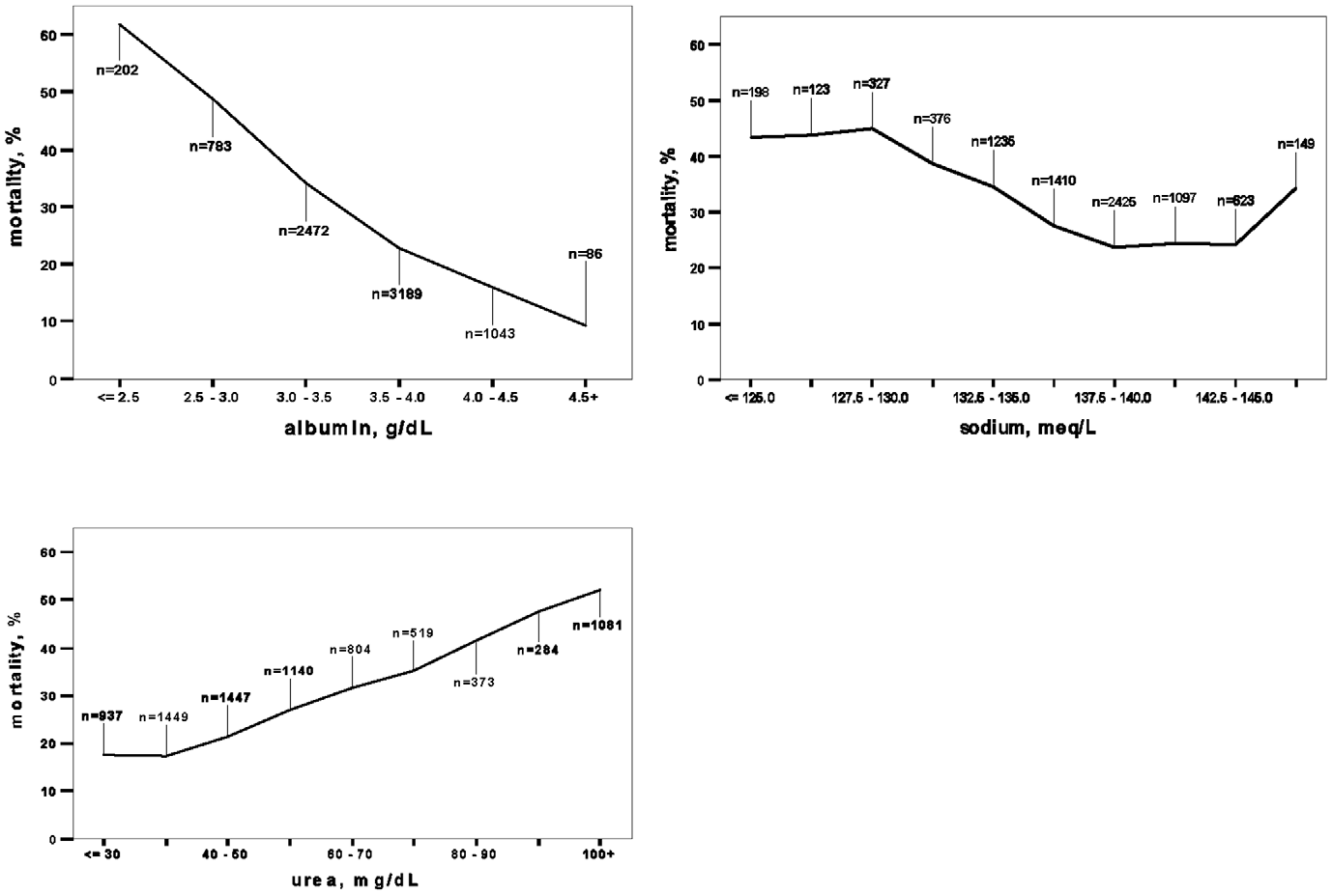
Results of the laboratory tests and one year mortality rates. Three laboratory tests with abnormal results associated with one-year mortality and highest odds ratio (albumin, sodium and urea).

**Figure 2:**
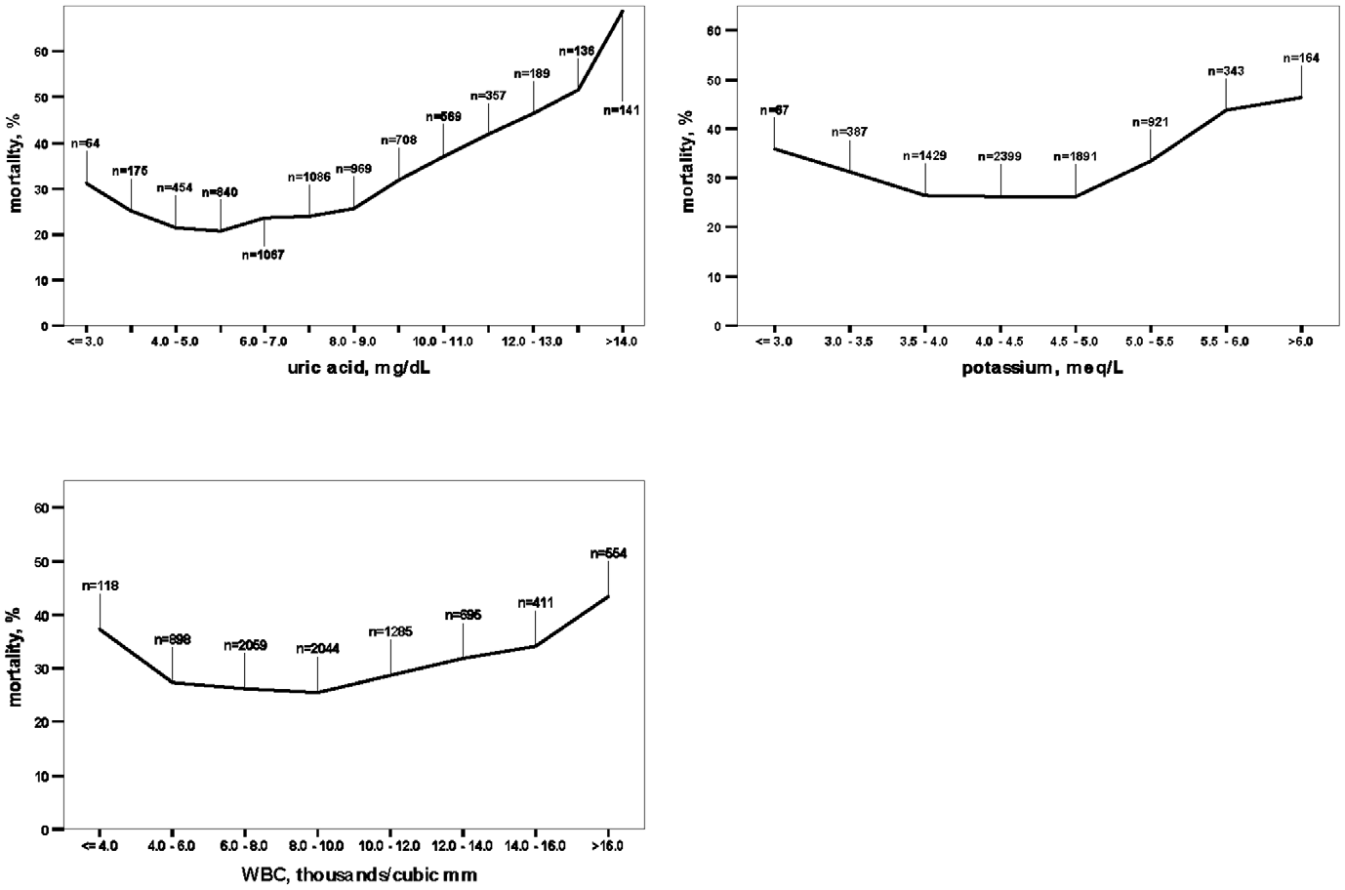
Results of the laboratory tests and one year mortality rates. Three laboratory tests with abnormal results associated with one-year mortality and lowest odds ratio (uric acid, potassium and leukocytosis).

During the first 24 hours of admission sodium levels were available for 7,963 patients (96.6%,) and potassium in 7,601 (92.2%, Figure 1, Figure 2). Hyponatremia was found in 1755 patients (22.0%) and 1,428 (14.8%) had hyperkalemia. One-year crude relative risk (RR) of death in patients with hyponatremia at admission was 1.54 (95%CI 1.43–1.66) and 1.40 (95%CI 1.29–1.52) for hyperkalemia.

Hematocrit results were available in 8,047 patients (97.6%). Anemia was found in 57.1% of the patients and was more frequent among males: 63.7% vs. 49.9% in female patients, p<0.001. Patients with anemia had a one-year mortality RR = 1.24 (95%CI 1.17–1.34) as compared to non-anemic group. White blood count was available in 8,064 cases (97.8%). Leukocytosis was found in 26.8% of the patients and was associated with one-year death: RR = 1.29 (95%CI 1.20–1.38).

Serum createnine results were available for 8,038 patients (97.5%). GFR less than 60 ml/min was found in 84.2%, while 25.2% of the patients had GFR<30 ml/min. Patients with GFR<30 ml/min had higher one-year mortality risk: RR = 1.62 (95%CI 1.51–1.73). Blood urea results were available in 8,034 (97.4%) patients. High levels were present in 5,648 (70.3%) and were associated with one-year mortality: RR = 2.26 (95%CI 1.90–2.26).

Hypoalbuminemia was found in 36.3% out of 7,775 tested patients and was associated with one-year mortality: RR = 1.85 (95%CI 1.72–1.98). Uric acid levels were available in 6,755 patients (81.9%). An elevated level of uric acid was associated with one-year mortality: RR = 1.42 (95%CI 1.30–1.56).

Glucose results were available for 8,028 patients (97.4%) and 1,897 (23.6%) had levels above 200 mg/dL. There was no association between glucose>200 mg/dL and one-year mortality neither in patients with diabetes (73.9% had glucose>200 mg/dL), nor without (27.3% had glucose>200 mg/dL): p = 0.49 and p = 0.17 respectively.

### Multivariable Analysis

**Figure 3:**
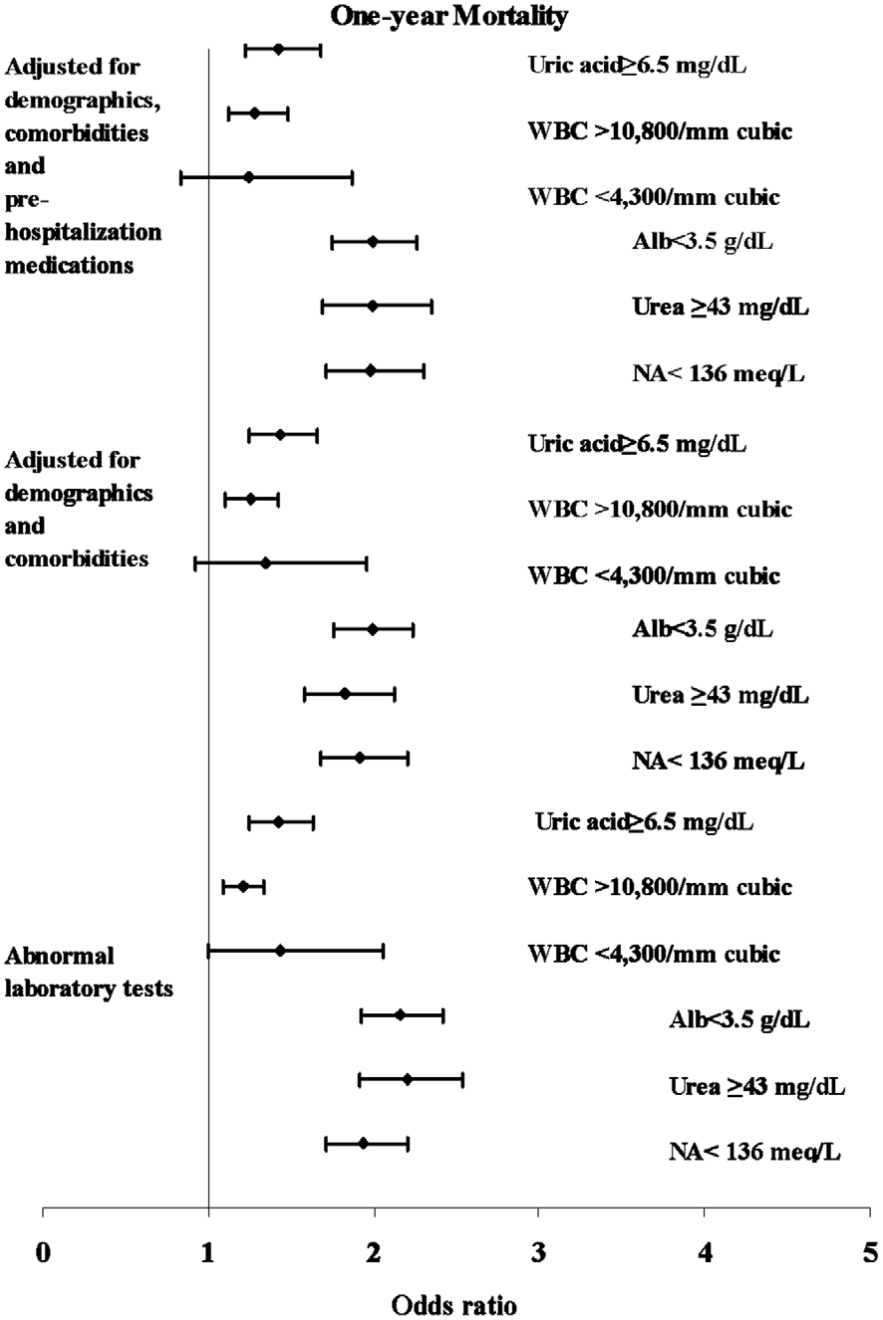
Odds ratios of laboratory values for 30-day all cause mortality. Five categorized laboratory tests found to be significant in the multivariable analysis as continuous variables are presented.

**Figure 4:**
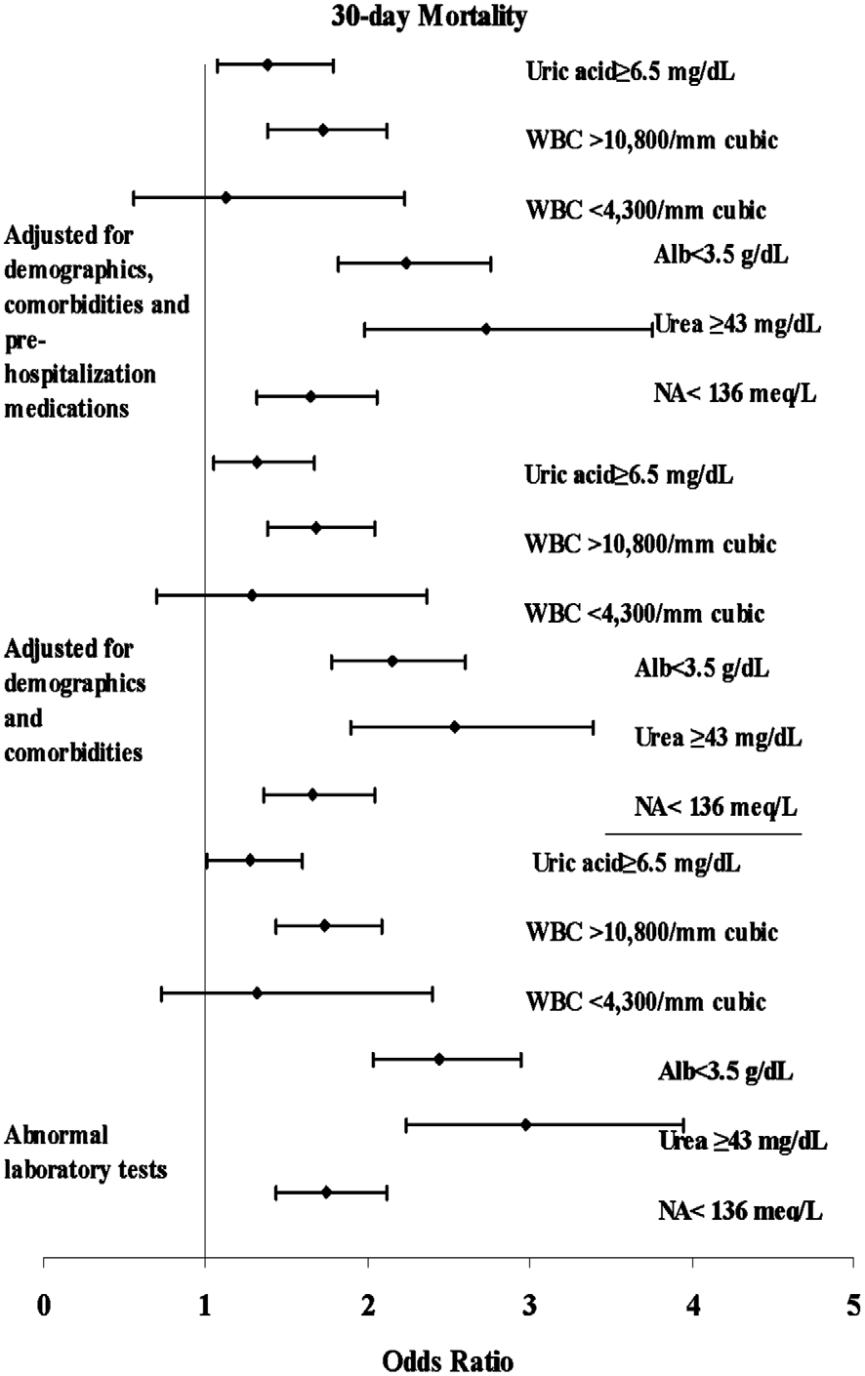
Odds ratios of laboratory values for one-year all cause mortality. Five categorized laboratory tests found to be significant in the multivariable analysis as continuous variables are presented.

Five laboratory test results: albumin, sodium, blood urea, uric acid and WBC were significantly related to 30-day and one-year mortality, as a group and individually, in unadjusted models as well as in models that adjusted for baseline clinical and demographic characteristics and for baseline medications. Corresponding odds ratios per one standard deviation increase are presented in Table 3. Generalized additive models revealed no meaningful departures from linearity for the laboratory variables. Hence, no new cut-points emerged. Figure 3 and Figure 4 presents odds ratios for laboratory values dichotomized using standard cut-points.

**Table 3:** Odds ratios* for five laboratory values calculated per standard deviation for sodium, blood urea, albumin and uric acid and for leucopenia and leukocytosis for WBC.

	One-year mortality			30-day mortality		
		95% Confidence Interval			95% Confidence Interval	
	Odds ratio	Lower limit	Upper limit	Odds ratio	Lower limit	Upper limit
Sodium	0.80	0.75	0.85	0.84	0.77	0.92
Blood urea	1.42	1.32	1.52	1.46	1.33	1.59
Albumin	0.64	0.60	0.69	0.63	0.57	0.69
WBC, normal range	Referent = 1.00					
WBC<4,300 10^3^/mm^3^	1.33	0.91	1.96	1.19	0.63	2.24
WBC>10,800 10^3^/mm^3^	1.23	1.08	1.41	1.60	1.31	1.96
Uric acid	1.31	1.23	1.40	1.31	1.19	1.44

*odds ratios expressed per one standard deviation increase in laboratory test level except for WBC which compares abnormal values to a reference group.

Derived from 30-day and one-year mortality prediction logistic regression models inclusive of laboratory tests, demographics and comorbidities.

### Discriminative and calibration abilities of the models for 30-day and one-year mortality prediction

**Figure 5:**
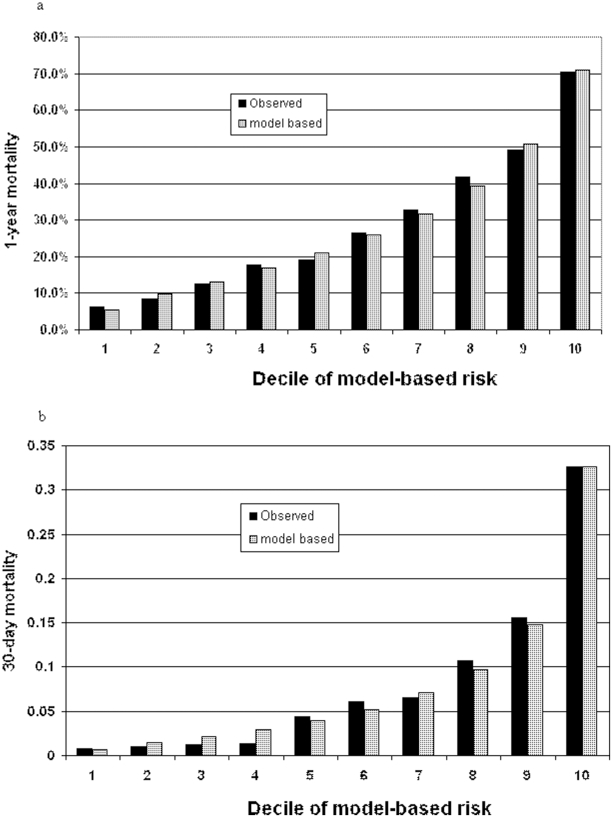
Calibration plot for 30-days and 1-year mortality. Model included laboratory tests, demographics and comorbidities.

C-statistics in the derivation cohort (3 hospitals, 3,941 subjects) and the validation cohort (4 hospitals, 4,305 subjects) were similar for all models and hence we decided to pool them and present results on combined data for increased precision. Table 4 presents the discriminative characteristics and calibration results for different combinations of variable groups (demographics, comorbidities, laboratory results and pre-hospitalization medications). Addition of the 5 laboratory test results to a model with baseline clinical and demographic characteristics increased the c statistic from 0.72 to 0.81 for 30-day mortality model and from 0.69 to 0.76 for one-year mortality model with both increases highly statistically significant (both p-values <0.0001). Figure 5 presents the calibration plot for the full model for 30-day and 1 year mortality prediction.

**Table 4:** Calibration and Discrimination of the logistic regression models.

		Demographics and comorbidities only		Laboratory tests only		Laboratory tests, demographics and comorbidities		Laboratory tests, demographics, comorbidities and pre-hospitalization medications*	
		c-statistics	Calibration chi-square and p-value	c-statistics	Calibration chi-square and p-value	c-statistics	Calibration chi-square and p-value	c-statistics	Calibration chi-square and p-value
**30-day mortality**		0.72	6.02	0.76	4.81	0.81	11.82	0.82	10.19
	p-value/C.I.	(0.70–0.74)	0.65	(0.74–0.78)	0.78	(0.79–0.83)	0.16	(0.78–0.84)	0.25
**One-year mortality**		0.69	7.63	0.72	9.78	0.76	6.61	0.77	4.35
	p-value/C.I.	(0.68–0.70)	0.47	(0.71–0.74)	0.28	(0.75–0.78)	0.58	(0.73–0.79)	0.82

*-adjusted for pre-hospitalization use of angiotensin-converting enzyme inhibitors, angiotensin receptors blockers, β-blockers, statins, spironolactone, digoxin and diuretics.

Laboratory tests include albumin, urea, sodium, uric acid and WBC.

Using the maximum sum of sensitivity and specificity as a rule to obtain the best cut-off point for classification we arrived at the following results:

For 1 year mortality the classification threshold was 0.28 with a sensitivity of 68.8% and specificity of 72.7%. Observed mortality rate for subjects classified as high risk was 48.2% (predicted mortality 47.7%) and as low risk 14.8% (predicted mortality 15.2%).

For 30-day mortality the classification threshold was 0.08 with a sensitivity of 73.1%% and specificity of 70.1%. Observed mortality rate for subjects classified as high risk was: 19.5% (predicted mortality 18.8%) and as low risk 3.0% (predicted mortality 3.3%).

### Echocardiography results

We included echocardiography data obtained within ±3 month from the index hospitalization for 854 subjects admitted to one of the hospitals. Severe systolic dysfunction (ejection fraction<30%) was found in 41.2% and diastolic dysfunction was diagnosed in 35.3% of the patients. Ejection fraction and diastolic dysfunction were added to demographic characteristics and comorbidities variables group. Inclusion of echocardiography results did not change the overall discriminative ability of the models or calibration: neither for 30-day, nor for one-year mortality prediction.

### Simplified clinical score

A simplified version of the clinical prediction score based on three dichotomized abnormal laboratory results (blood albumin, sodium and urea, available for 7,608 patients, 92.3%) was developed. One-year logistic regression model inclusive of uremia, hypoalbuminemia, hyponatremia, demographics and comorbidities yielded the following odds ratios: uremia 1.50; hypoalbuminemia 1.73 and hyponatremia 1.63. Since the odds ratios were similar, each abnormal result was assigned 1 point. The score ranged from 0 points (1,300 patients, 17.1%), 1 point (3,397 patients, 44.7%), 2 points (2,338 patients, 30.7%) to 3 points (573 patients, 7.5%) and was capable of discriminating from 1.4% to 20.2% in 30-day mortality and 11.2% to 55.0% in one-year mortality rates between patients with a score of 0 to 3. Table 5 shows that mortality risk at 30-day and one-year were similar across the entire spectrum of risk between derivation and validation cohorts.

**Table 5:** Mortality rates stratified by the score.

		Score			
		0 points	1 point	2 points	3 points
**30-day mortality rate**	derivation cohort	1.0%	5.1%	13.0%	17.8%
	validation cohort	1.7%	6.7%	13.5%	23.4%
**1-year mortality rate**	derivation cohort	11.2%	23.6%	39.9%	55.3%
	validation cohort	12.0%	25.0%	40.0%	55.9%

For each abnormal result in admission laboratory test (urea≥43 mg/dL, sodium<136 meq/L and albumin<3.5 g/dL) one point was assigned. Patient population was stratified by the total combined score. Mortality rates presented for derivation cohort (3 hospitals, 3,941 subjects) and validation cohort (4 hospitals, 4,305 subjects).

Missing score rates were distributed evenly between the hospitals being less than 10% in each one of them (in total missing in 7.7% patients). Thirty day mortality was slightly higher in group of patients with missing score (10.5% vs. 8.3%) while 1-year mortality was slightly lower (27.7% vs. 28.8%).

**Figure 6:**
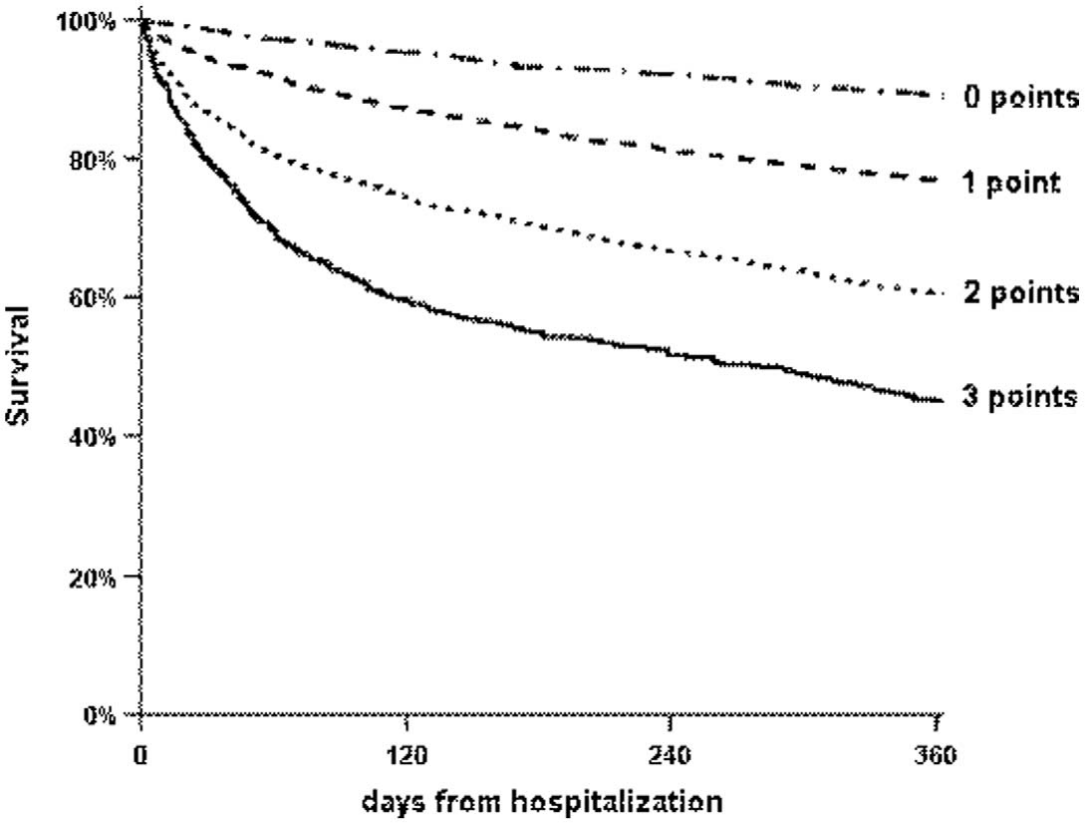
Kaplan-Meier curves for one-year mortality according to risk points. For each abnormal result in admission laboratory test (urea≥43 mg/dL, sodium<136 meq/L and albumin<3.5 g/dL) one point was assigned. Patient population was stratified by the total combined score. Log-rank test, p<0.001.

Length of the hospitalization increased with higher score: from a median of 3 days (IQR 2–5) in group of patients with score of 0, 4 days (IQR 2–6) in score 1, 4 days (IQR 3–8) in score 2 and 5 days (IQR 3–10) in patients with score 3, p<0.001 for trend. Kaplan-Meier analysis (figure 6) showed that assignment of the risk score at baseline maintained the prognostic implication at all time point up to 1 year (log-rank test p<0.001).

## Discussion

Assessing the prognosis of HF patients at admission is essential for several reasons. It can help determine the intensity of initial treatment and monitoring, facilitate triage decisions, provide initial information to patients and relatives, and is also important for audit and retrospective quality control.

We found that the addition of readily available laboratory tests improve significantly the predictive ability of the survival models based on clinical and demographic characteristics. A few routine test results (albumin, urea, WBC, uric acid and sodium) have a discriminative ability for 30-day and one-year mortality predictions comparable with more sophisticated and expensive biomarkers such as BNP, NT-proBNP, H-FABP, Mid-Regional pro-Adrenomedullin (MR-proADM) and PTX3.[17], [18], [19]

Several scores were proposed for predicting survival in these patients, but they vary in the choice, extent and availability of the required components. Lee et al [24] developed a score composed of eleven variables, including demographic characteristics, co-morbid conditions, clinical signs and selected laboratory values obtained within twenty four hours of admission. The overall prediction accuracy for this score is similar to the discriminative power of our full models (demographic characteristics, comorbidities and laboratory tests results): c-statistics for 30-days 0.79 vs. 0.81 and for one-year mortality 0.76 vs. 0.76 respectively. However, it requires data on vital signs such as blood pressure and respiratory rates. The Heart Failure Survival Score [HFSS] requires more elaborate calculation and stratifies patients with NYHA class III and IV based on history, heart rate, LVEF, BP, IVCD, sodium and peak VO_2_.[25] More recently, The Seattle Heart Failure Model was developed which uses a combination of history, ejection fraction, BP, medication use and laboratory values to predict survival for ambulatory and hospitalized patients with HF.[26] Using the ADHERE registry, Fonarow et al. was able to risk stratify patients into low, intermediate and high risk representing a 2–22% range for in hospital mortality using levels of urea, createnine and SBP with an area under the curve of 0.67–0.69. [27]

As there is a clear trade-off between the simplicity and ease of use of available prediction tools and the accuracy of prediction, the challenge is to choose an optimal tool for the task. Three of the easily obtainable routine admission tests (albumin, sodium and urea) can constitute a simple prognostic score with one point assigned for each abnormal result. This score was able to risk stratify 30-day mortality with a range of 1%–21% and one-year mortality in a range of 12–56%. Compared with the full model and other similar survival prediction tools this short score, based on the standard cut-off point for the normal range of the routine laboratory tests, allows for rapid implementation on the level of the treating physician. Its simplicity eliminates the need for elaborate calculations requiring bed-side computers or memorization of the different schemes for the score point assignment.

Our study does not necessarily present new information on the potential mortality predictors in patients with acute decompensated heart failure. Rather we have shown that the use of simple and relatively inexpensive laboratory tests may achieve the same degree of accuracy in predicting the outcomes as more contemporary and sophisticated biomarkers. One can assume that the biomarkers are not independent from the patient's clinical condition. The severity of the disease can be evident by analyzing number of the patient's clinical signs and symptoms as well as laboratory tests. The added value of the individual test is usually small (though sometimes important); therefore the approach based on the entire clinical picture might provide a greater insight into the individual patient prognosis. Moreover, the likelihood of survival can be determined reliably only in populations and not in individuals.[28]

Our study has a number of limitations. We did not have a direct comparison to discriminative abilities of more novel biomarkers such as BNP/NT-proBNP. BNP/NT-proBNP have been shown to be predictive of mortality in several studies of HF, however these tests are not routinely available in many countries and add significantly to laboratory costs.[27], [29] The study was only performed in one country; however the care of patients with HF in Israel has been shown to be similar to that of European and North American countries.[30] Patients included in this retrospective study were those with a discharge diagnosis of HF as recorded by the treating physicians and coded with ICD-9 classification without consistent capturing of patients' functional status (NYHA class). We were not able to separate between the first incidence of HF admission in life and repeated admission. It might be expected that repeated admission patients have different prognosis. Our analysis included data from the hospitals operating under the centrally managed universal health system (Clalit Health Services), therefore it might be expected that the models will perform differently in other countries. Lastly, although the participating hospitals belong to the same health care system, some variations in laboratory measurements might exist.

The strength of our study is the large number of consecutive, non-selected patients studied and the applicability of the score to all patients presenting to the hospital with a diagnosis of HF. In addition we had the complete follow-up data on long term mortality. Our analysis shows that based on the administrative data analysis (patient demographics, recorded diagnosis and number of laboratory tests results) the reasonable degree of the prognostication can be achieved.

In summary, we have confirmed that a number of demographic characteristics, comorbidities and abnormal laboratory tests are predictive of 30-day and one-year mortality in patients admitted with decompensated HF. A small panel of easily obtainable laboratory tests can help risk-stratify patients with HF. These results need to be prospectively confirmed in other cohorts of HF patients.
